# Getting the chemistry right: protonation, tautomers and the importance of H atoms in biological chemistry

**DOI:** 10.1107/S2059798316020283

**Published:** 2017-02-01

**Authors:** Ben Bax, Chun-wa Chung, Colin Edge

**Affiliations:** a Structural Biology, MRC Laboratory of Molecular Biology, Francis Crick Avenue, Cambridge CB2 0QH, England; b Platform Technology and Science, GlaxoSmithKline, Medicines Research Centre, Gunnels Wood Road, Stevenage SG1 2NY, England

**Keywords:** tautomers, chemistry, H atoms, ligands

## Abstract

There are more H atoms than any other type of atom in an X-ray crystal structure of a protein–ligand complex, but as H atoms only have one electron they diffract X-rays weakly and are ‘hard to see’. The positions of many H atoms can be inferred by our chemical knowledge, and such H atoms can be added with confidence in ‘riding positions’. For some chemical groups, however, there is more ambiguity over the possible hydrogen placements, for example hydroxyls and groups that can exist in multiple protonation states or tautomeric forms. This ambiguity is far from rare, since about 25% of drugs have more than one tautomeric form. This paper focuses on the most common, ‘prototropic’, tautomers, which are isomers that readily interconvert by the exchange of an H atom accompanied by the switch of a single and an adjacent double bond. Hydrogen-exchange rates and different protonation states of compounds (*e.g.* buffers) are also briefly discussed. The difference in heavy (non-H) atom positions between two tautomers can be small, and careful refinement of all possible tautomers may single out the likely bound ligand tautomer. Experimental methods to determine H-atom positions, such as neutron crystallography, are often technically challenging. Therefore, chemical knowledge and computational approaches are frequently used in conjugation with experimental data to deduce the bound tautomer state. Proton movement is a key feature of many enzymatic reactions, so understanding the orchestration of hydrogen/proton motion is of critical importance to biological chemistry. For example, structural studies have suggested that, just as a chemist may use heat, some enzymes use directional movement to protonate specific O atoms on phosphates to catalyse phosphotransferase reactions. To inhibit ‘wriggly’ enzymes that use movement to effect catalysis, it may be advantageous to have inhibitors that can maintain favourable contacts by adopting different tautomers as the enzyme ‘wriggles’.

## Abstract 2

H atoms are ‘hard to see’ in X-ray crystal structures of protein–ligand complexes. This paper discusses the problem of identifying the correct tautomeric form(s) of protein-bound ligands.

## 1. Introduction   

The most famous story about tautomers in the history of science occurred in the early 1950s in Cambridge. Watson and Crick were trying to propose a structure for DNA, but had been failing for some time. They were, however, fortunate enough to be sharing an office with the American theoretical chemist Jerry Donahue. One Wednesday afternoon they discussed the possible tautomeric forms of the bases in DNA. Jerry Donahue told Watson and Crick that the literature was likely to be wrong and what the most probable tautomers for G, C, A and T were. When Jim Watson came in to work at 9.30 am on Saturday morning he had cardboard models for the four bases in the ‘correct’ tautomeric forms and, by the time that Francis Crick arrived for work at 10.30 am, Jim had worked out the classical G–C, A–T base pairing (J. Watson seminar, LMB, Cambridge, 9th June 2016). As they subsequently wrote in their famous paper

**Figure 1:**
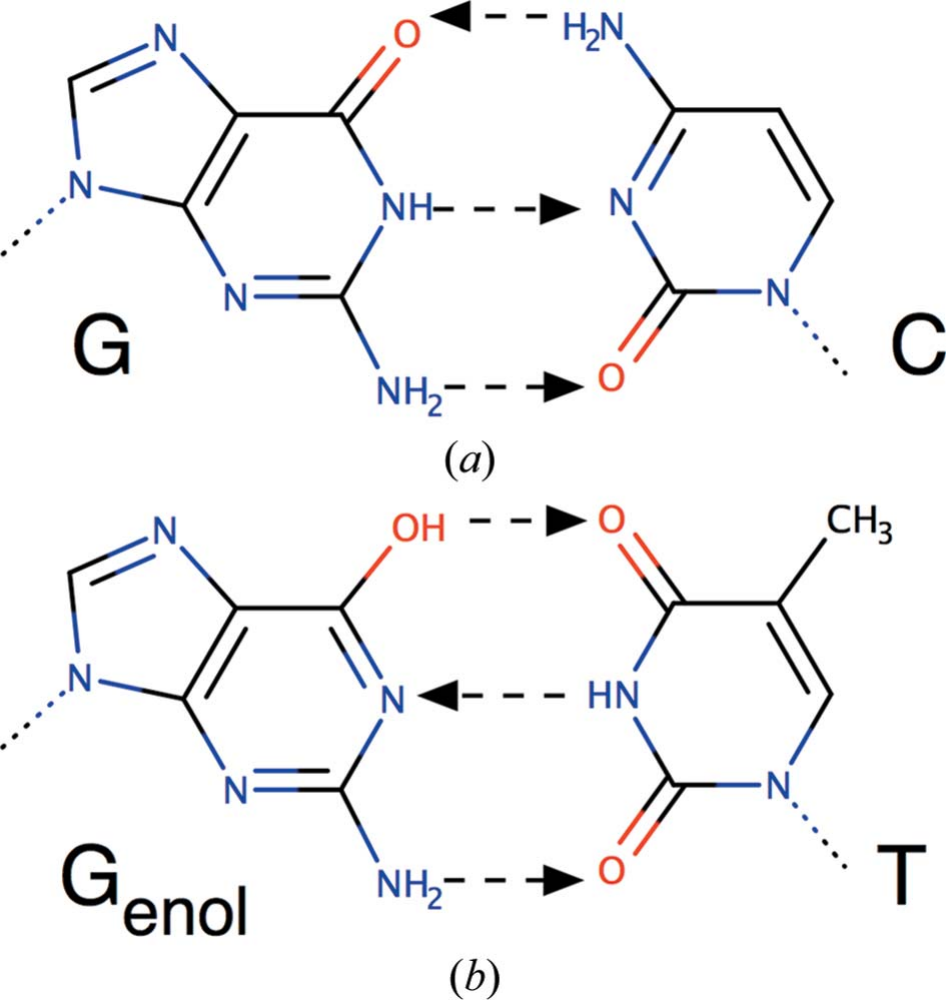
Comparison of (*a*) the classical ‘Watson–Crick’ G–C base pair with (*b*) a G–T base pair that guanine can make when it adopts a less stable enol tautomer (dashed arrows from hydrogen-bond donor to acceptor) (Topal & Fresco, 1976 ▸). *Marvin* was used to draw chemical structures (https://www.chemaxon.com).

If it is assumed that the bases only occur in the most plausible tautomeric forms … it is found that only one specific pair of bases can bond together

(Watson & Crick, 1953 ▸). The normal Watson–Crick base-pairing for G–C is shown in Fig. 1 ▸, which also shows an unusual G–T base pair that could be made if the guanine adopted an enol tautomer (Topal & Fresco, 1976 ▸). This story illustrates that understanding tautomers can be important in understanding molecular-recognition processes, and also how valuable it can be to know a good chemist.

In an enumeration of 1791 marketed drugs, 74% existed only as one tautomer, while 26% existed as an average of three tautomers (Martin, 2009 ▸). In principle, it is possible to experimentally determine the positions of H atoms/protons in a ligand–protein complex, with the technique of choice being neutron diffraction (Kwon *et al.*, 2017 ▸). While a limited number of high-resolution neutron structures of ligand complexes do exist in the PDB (Blakeley, 2016 ▸; Fisher *et al.*, 2012 ▸), a 2015 survey showed there were only 83 structures with neutron data in the PDB, compared with over 90 000 X-ray crystal structures (Blakeley *et al.*, 2015 ▸), perhaps reflecting the greater technical difficulties in determining neutron structures (Kwon *et al.*, 2017 ▸).

This paper focuses on strategies to address the problem of how to determine which tautomeric (or protonation) state your ligand is in when you have determined an X-ray crystal structure of the complex. It is meant to serve as a brief introduction and reminder to structural biologists of the importance of H atoms in biological chemistry. The paper has sections on ‘Tautomers, protonation states and hydrogen exchange’ (§[Sec sec2]2) and ‘Using small-molecule crystal structures to define ligand chemistry’ (§[Sec sec3]3), followed by a brief discussion of ‘When and how to add H atoms to your ligand protein complex?’ (§[Sec sec4]4). Two examples of fitting tautomers, AMPPNP and QPT-1, into macromolecular X-ray crystal structures are then discussed (§[Sec sec5]5). The paper concludes with a brief outline of some ‘Experimental techniques to try to determine where your H atoms are’ (§[Sec sec6]6) and ‘Conclusions’ (§[Sec sec7]7).

## 2. Tautomers, protonation states and hydrogen exchange   

### 2.1. Tautomers   

**Figure 2:**
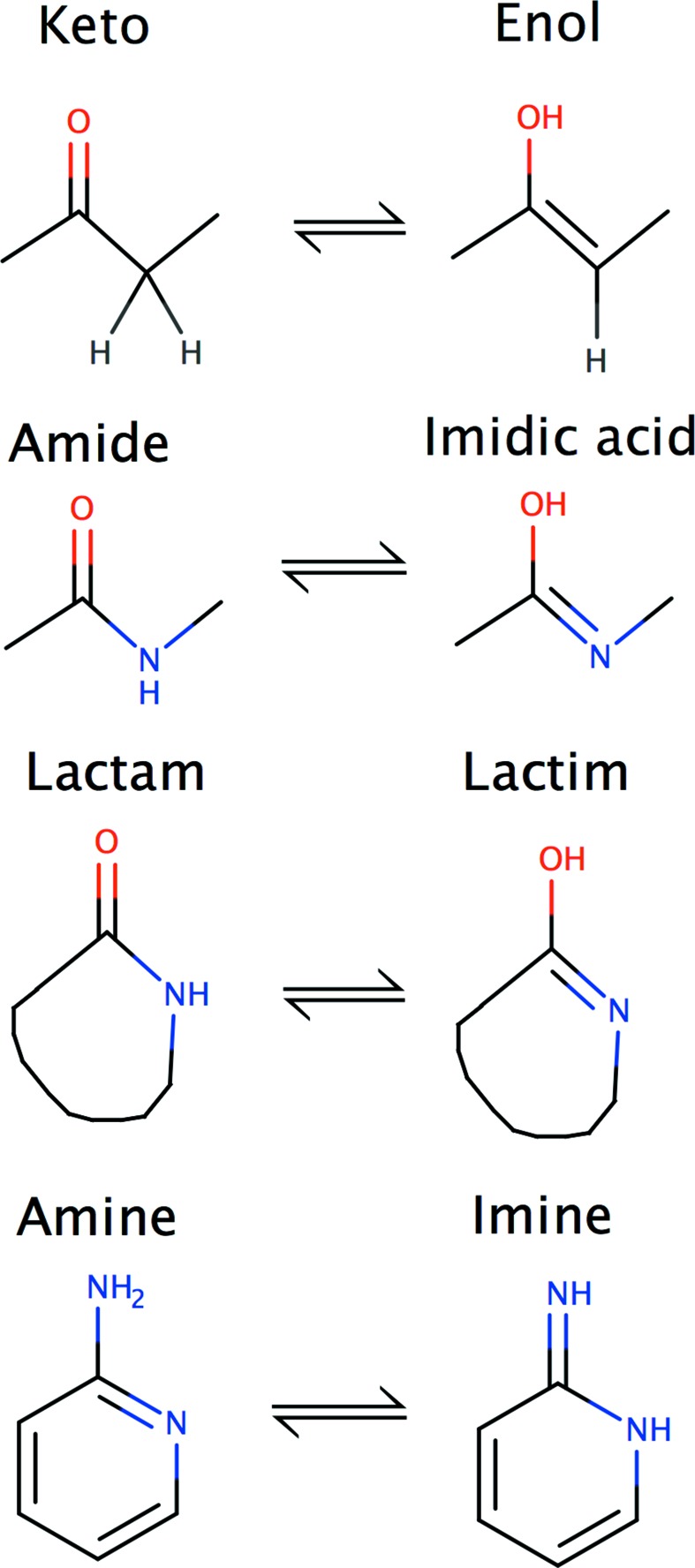
Four common types of tautomerization: keto–enol, amide–imidic acid, lactam–lactim and amine–imine. Note that a lactam is a cyclic amide. *Marvin* was used to draw chemical structures (https://www.chemaxon.com).

Tautomers are compounds that readily interconvert by the ‘movement of an atom (usually hydrogen) or group of atoms from one site to another within the molecular structure’ (Katritzky *et al.*, 2010 ▸). It should be noted that there is no clear dividing line between isomers and tautomers: ‘tautomers are simply isomers that convert with a relatively low activation energy below 20 kcal mol^−1^’ (Katritzky *et al.*, 2010 ▸). The focus of this paper is on the commonest types of tautomer, those that involve the formal migration of an H atom or proton, accompanied by a switch of a single bond and an adjacent double bond (Fig. 2 ▸). Ring–chain tautomers, which can play important roles in isomerization of sugars (Zhu *et al.*, 2001 ▸) and also occur in warfarin (Martin, 2009 ▸; Supplementary Figure S1), will not be discussed further in this paper. Tautomers in which a C—H bond is cleaved or formed (such as the keto–enol tautomers shown in Fig. 2 ▸) can sometimes be isolated as separate species because breaking or forming a C—H bond is a relatively slow process (Katritzky *et al.*, 2010 ▸). In contrast, tautomers which involve the exchange of H atoms between polar atoms (such as amide–imidic acid) are usually very rapid (exposed main-chain amide H atoms exchange about 100 times per minute at pH 7).

### 2.2. Hydrogen exchange   

**Figure 3:**
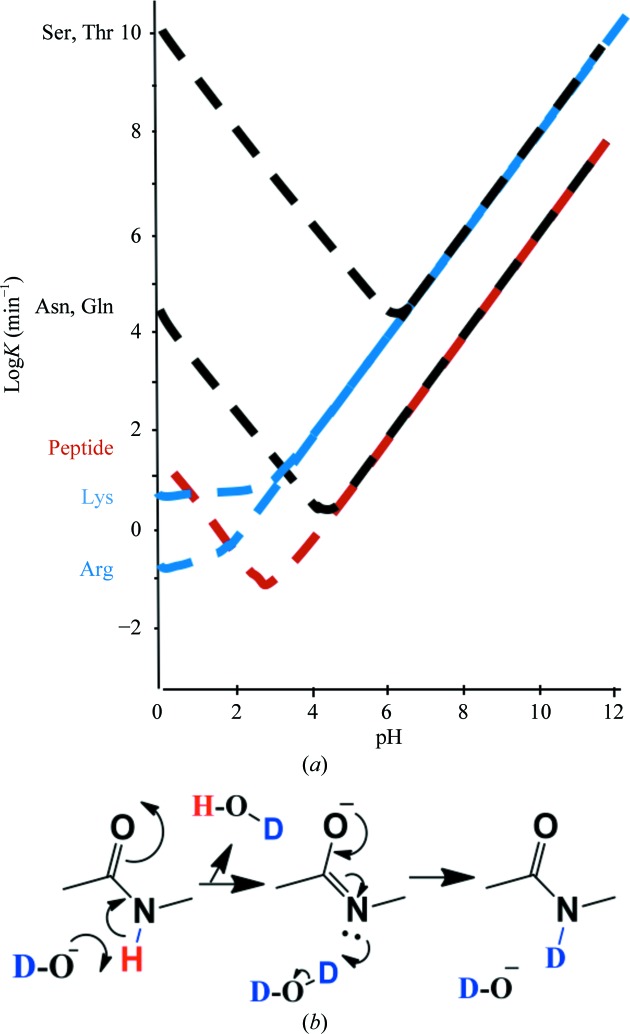
(*a*) Measured exchange rates of some labile protons in a protein (BPTI) *versus* pH (adapted from Wüthrich & Wagner, 1979 ▸). (*b*) Base-catalyzed peptide hydrogen–deuterium exchange is likely to proceed *via* an imidic acid intermediate. *Marvin* was used to draw chemical structures (https://www.chemaxon.com).

**Figure 4:**
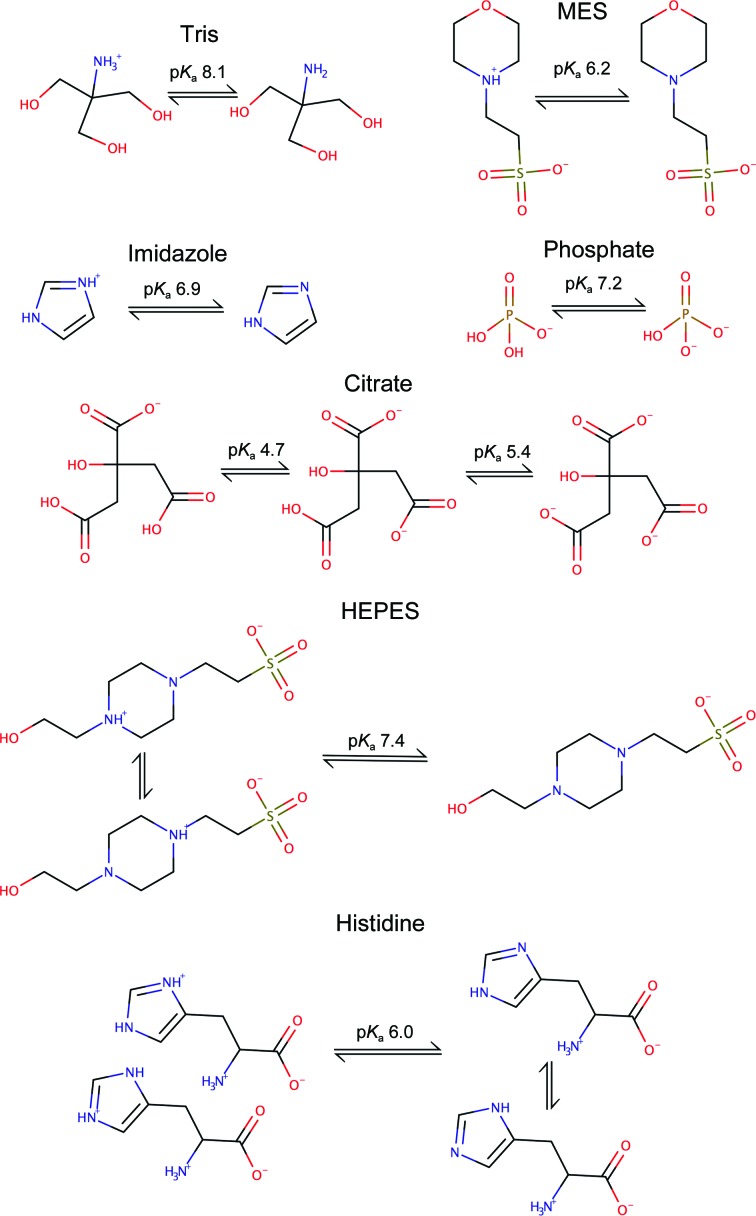
Alternative protonation states of some common buffers (note the alternative tautomeric forms of HEPES at lower pH and histidine at higher pH). *Marvin* was used to draw chemical structures (https://www.chemaxon.com).

H atoms attached to polar (N and O) atoms that are exposed to aqueous solvent usually exchange very rapidly. Fig. 3 ▸ shows the exchange rates of labile protons in the small protein BPTI (Wüthrich & Wagner, 1979 ▸). In studying protein structure using hydrogen–deuterium exchange experiments, main-chain N—H exchange is quenched (but not entirely eliminated) by lowering the pH to 2.8 (Fig. 3 ▸ *a*). The base-catalysed exchange of the main-chain N—H group probably proceeds *via* an imidic acid intermediate (Fig. 3 ▸ *b*). The side chain of a histidine residue has two tautomers when it is not protonated (Fig. 4 ▸, bottom right), while at lower pH both N atoms on the imidazole ring are protonated and the positive charge can be stabilized around the aromatic ring (Fig. 4 ▸, bottom left). The two Kekulé representations of the positively charged histidine side chain (Fig. 4 ▸, bottom left) are inadequate (Katritzky *et al.*, 2010 ▸) as resonance will stabilize the positive charge around the aromatic imidazole ring.

### 2.3. Protonation states   

The most common ligands that structural biologists encounter that have alternative protonation states are buffers. Fig. 4 ▸ shows protonation equilibria of some common buffers. Note that below pH 7.4 HEPES [(4-(2-hydroxyethyl)-1-piperazineethanesulfonic acid] is a zwitterion, with a negative charge on the sulfate and a positive charge on one of the N atoms on the central piperazine ring. In the zwitterionic form the protonated N atom on the piperazine ring can act as a hydrogen-bond donor, while the unprotonated N atom can act as a hydrogen-bond acceptor. The protonation states shown in Fig. 4 ▸ are only those that occur at common pH values. In the active site of an enzyme unusual protonation states may be observed; for example, in a joint X-ray and neutron diffraction study of metal-ion roles and the movement of hydrogen during a reaction catalysed by d-xylose isomerase, Kovalevsky *et al.* (2010 ▸) observed that ‘Lys289 is neutral before ring opening but gains a proton after this’.

## 3. Using small-molecule crystal structures to define the ligand chemistry of tautomers   

### 3.1. Generating restraint dictionaries for tautomers   

**Figure 5:**
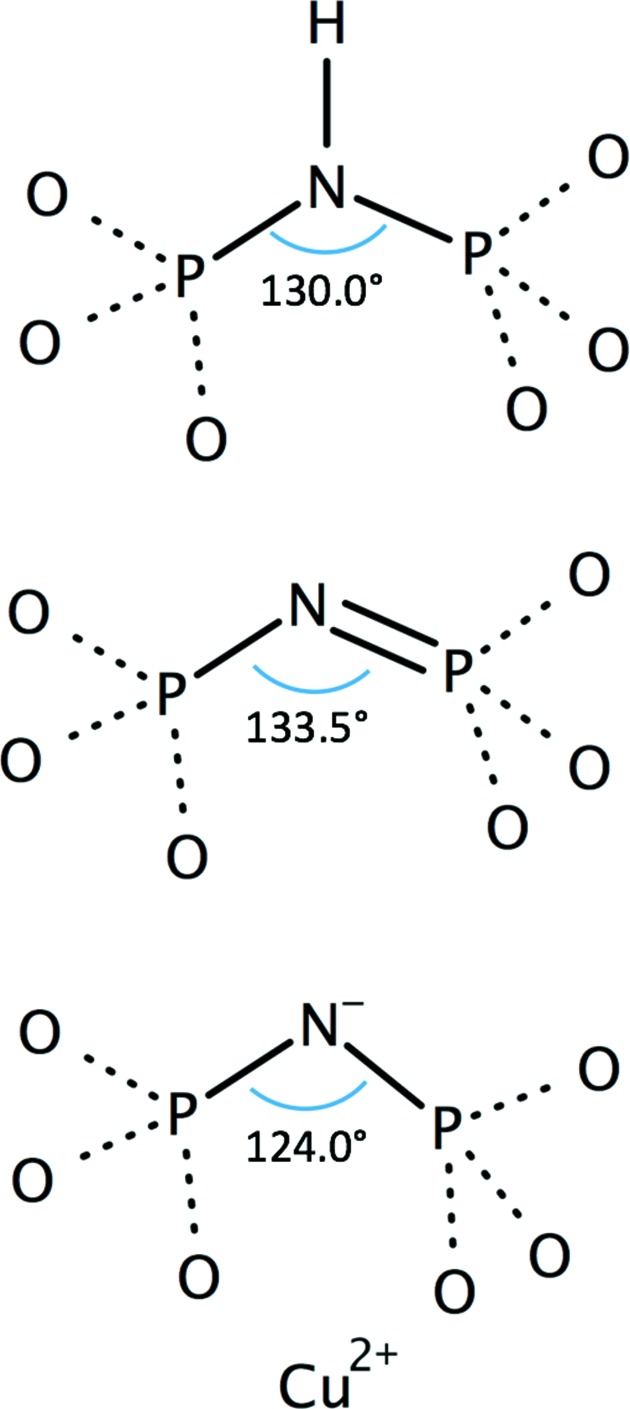
P—N—P bond angles derived from the CSD. The dotted lines between P and O atoms indicate that any bond type was allowed in the search of the CSD with *ConQuest*. Structures in the CSD which have a metal ion coordinated by two of the phosphate O atoms have a more acute P—N—P bond angle, presumably because this brings the two O atoms coordinating the metal closer together. *Marvin* was used to draw chemical structures (https://www.chemaxon.com).

Most modern programs for generating restraint dictionaries for ligands (Steiner & Tucker, 2017 ▸; Long *et al.*, 2017 ▸) use small-molecule crystal structures either from the CSD (Cambridge Structural Database) or the COD (Crystallo­graphy Open Database). Small-molecule crystal structures are also a valuable source of information for the study of tautomers. Although automated ligand-restraint generation can give excellent dictionaries (Steiner & Tucker, 2017 ▸), it can be informative to look at the crystal structures from which the restraints are generated. Structures in the CSD can easily be found and examined with the program *ConQuest* (Bruno *et al.*, 2002 ▸). Fig. 5 ▸ and Table 1 ▸ give an example of the different types of geometry that examination of the CSD with *ConQuest* suggests for a PO_3_—N—PO_3_ or PO_3_—NH—PO_3_ geometry. Manual examination of the structures suggested that the geometry of the P—N—P bond may be influenced by the presence of a metal ion coordinated by two of the O atoms on the phosphates (Fig. 5 ▸ and Table 1 ▸); automated programs do not always spot such subtleties. Sometimes it is necessary to edit an initial refinement dictionary so that it conforms to the chemistry of the required tautomer(s).

### 3.2. Identifying questionable tautomers in small-molecule crystal structures   

One of the reasons that Watson and Crick needed Jerry Donahue’s advice was because a small-molecule crystal structure of a guanine in the literature was in an unusual tautomer, and Jerry knew from quantum-mechanical calculations in the literature that the tautomer in the crystal structure was ‘just a guess’. Today (2016) diffraction data extending to at least 0.83 Å resolution are required for the publication of a small-molecule crystal structure in *Acta Crystallographica Section C*, and at this resolution nearly all H atoms in small-molecule crystal structures are visible. However, H atoms often need to be refined with restraints (Sheldrick, 2015 ▸), and questionable tautomers do still occasionally appear in the Cambridge Structural Database (CSD). In a study of tautomers in the CSD, Cruz-Cabeza & Groom (2011 ▸) showed that simple quantum-mechanical calculations could be used to identify implausible tautomers where there were unusually short contacts or large differences in energies between observed and putative tautomers. Interestingly, only some 10% of the molecules in the CSD were predicted to have tautomers, and only 0.5% of these were actually observed as different tautomers in the CSD (Cruz-Cabeza & Groom, 2011 ▸).

*Mogul*, a CCDC program (Bruno *et al.*, 2004 ▸), can also be used to help identify discrepant geometries that imply incorrectly modelled tautomers. *Mogul* can be used to check small-molecule crystal structures. It compares bond lengths, bond angles, torsion angles and some ring angles with those of similar molecules found in the CSD and highlights features that are uncommon and perhaps incorrect. In some cases this is sufficient to identify misassigned hydrogen positions. Three examples of the use of *Mogul* to distinguish between pairs of implausible/plausible tautomers are given below. Cruz-Cabeza & Groom (2011 ▸) point to the structures of 3-chloro-1,2,4-triazole, where *Mogul* can distinguish between the dubious model configuration with CSD refcode CLTRZL and the more plausible CSD refcode CLTRZL01 (Claramunt *et al.*, 2001 ▸; Supplementary Fig. S2*a*). A more recent example is a comparison of the 1,3-thiazol-4-one structures with CSD refcodes GACXOZ and LOQBIE (Gzella *et al.*, 2014 ▸), in which *Mogul* queries the C—N bond length of the imine of GACXOZ, but finds all geometrical parameters of the amine version to be within expected limits (Supplementary Fig. S2*b*). Unfortunately, *Mogul* does not always provide a definitive answer, as in the case of a comparison of the 2-amino-1,3,4-thiadiazole configurations with CSD refcodes UKIRAI and UKIRAI02 (Li *et al.*, 2014 ▸), where both configurations are deemed to have unusual geometries, although UKIRAI does have more questionable features (highlighted in red in Supplementary Fig. S2*c*) than UKIRAI02. *Mogul* is a recommended first step in assessing the atomic configuration of ligands, along with visual inspection of the modelled geometry.

## 4. When and how to add H atoms to your ligand complex?   

Macromolecular crystal structures can be refined with or without riding H atoms. However, when you deposit your structure with the PDB, part of the structure-validation process (Gore *et al.*, 2012 ▸) is to add H atoms to the protein (with *Reduce*; Word *et al.*, 1999 ▸) and to then check them with *MolProbity* (Chen *et al.*, 2010 ▸; Deis *et al.*, 2013 ▸). Ligand-validation programs (Adams *et al.*, 2016 ▸; Emsley, 2017 ▸) will also check for clashes between the ligand and the ligand-binding pocket once both have been protonated. However, most modern refinement programs have refinement terms (Steiner & Tucker, 2017 ▸) that try to eliminate unfavourable van der Waals contacts between H atoms. If your ligand can have multiple tautomeric states or protonation states, it can be useful to try and dock and refine all possible tautomeric states and protonation states into the binding sites. For example (see below and Chan *et al.*, 2015 ▸) we read SMILES (Weininger, 1988 ▸) strings for eight tautomers of QPT-1 into an *AFITT* (Wlodek *et al.*, 2006 ▸) script, and automatically docked each of the eight into six binding sites. Computational chemistry programs such as *MarvinSketch* (*Marvin* v.16.8.15, ChemAxon; https://www.chemaxon.com) can be used to enumerate possible tautomeric and charged states.

The procedure that we recommend for trying to see if you may have fitted the ‘wrong’ prototropic tautomer or proton­ation state of your ligand in a complex is as follows.

**Figure 6:**
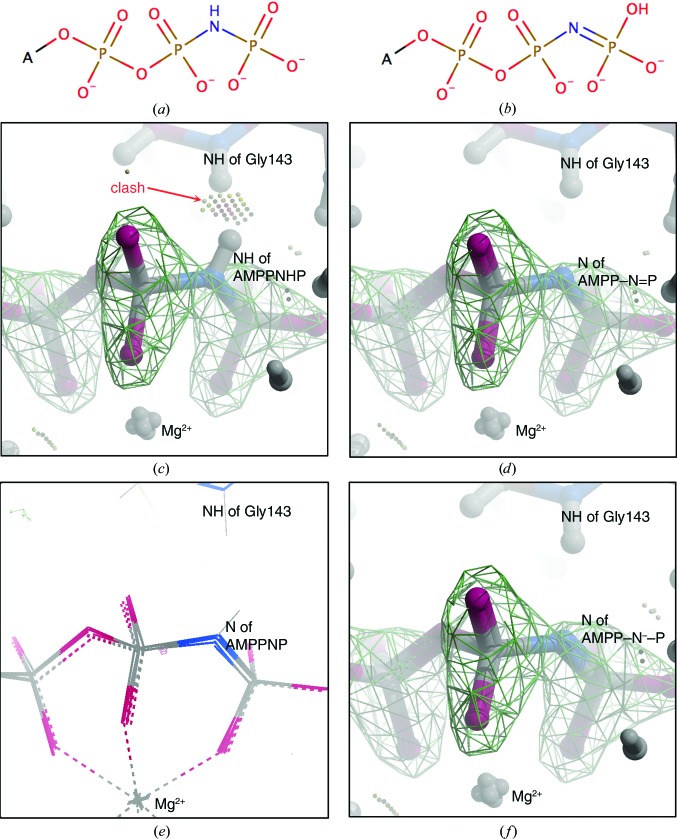
Comparison of the fitting of (*a*) AMPP—NH—P or (*b*) AMPP—N=P into a structure. In (*c*) the imido (P—NH—P) form was fitted into an *F*_o_ − *F*_c_ ligand-omit map (shown at 6.5σ), while in (*d*) and (*f*) the imino (P—N=P or P—N**^−^**—P) form was fitted into the same map (see Table 1 ▸ for restraints). Small overlaps and bad overlaps are displayed as dots. (*e*) shows that there are only small differences between the fitted coordinates from (*c*), (*d*) and (*f*) and the deposited structure (PDB entry 1pvg; the four structures are shown superposed). (*a*) and (*b*) were drawn with *Marvin* (https://www.chemaxon.com) and (*c*), (*d*), (*e*) and (*f*) with *Coot* (Emsley *et al.*, 2010 ▸).

(i) Refine and fit your ligand to the density as you would normally do. Quite often differences between tautomers are quite ‘small’, so which tautomer you fit initially may not be that important. If you have fitted the ‘wrong’ tautomer you might think that you would see a clash of H atoms; however, unless you have very high resolution data the refinement program will often slightly adjust the conformation of both the protein and the ligand to avoid such ‘hydrogen’ clashes.
(ii) Delete the ligand from the ‘completed final’ structure and refine for a few rounds to allow the protein to ‘relax’ into its ‘correct’ conformation and give the ‘best’ possible difference map to fit the ligand into.
(iii) Fit all possible tautomers/protonation states of your ligand into this ‘best’ difference map (look at each carefully on the graphics to check that it is fitted reasonably into the density) and then refine each possible solution.
(iv) A final validation check should be made for all possible solutions, including checking of ligand geometry in the refined structure(s) with *Mogul* (Bruno *et al.*, 2004 ▸) and careful examination of maps in *Coot* (Emsley *et al.*, 2010 ▸). The interactions between the ligand and the protein can be examined in *Coot* with the ‘Ligand’→‘isolated dots for this ligand’ command (Emsley, 2017 ▸): this gives a *MolProbity*-like view of contacts (including clashes) between the protein and the ligand (see, for example, Fig. 6 ▸).

## 5. Two examples of tautomers in macromolecular X-ray crystal structures   

### 5.1. Refining AMPPNP in the ATPase domain of a type IIA topoisomerase   

ATP has two common protonation states in solution, ATP^3−^ and ATP^4−^ (Alberty & Goldberg, 1992 ▸), which differ only in the presence or absence of an H atom on one of the O atoms on the γ-phosphate (Supplementary Fig. S3); the presence of an adjacent Mg^2+^ ion tends to shift ATP to the ATP^4−^ form. In AMPPNP the O atom between the β-phosphate and γ-phosphate of ATP is replaced by an N atom (Supplementary Fig. S3). In solution the N atom between the β-phosphate and γ-phosphate is normally protonated and the compound is known as adenosine-5′-(β,γ-imido)triphosphate (AMPP—NH—P). In some crystal structures of AMPPNP with proteins this bridging N atom accepts hydrogen bonds and is in the unprotonated imino form: adenosine-5′-(β,γ-imino)triphos­phate (AMPP—N=P) (Dauter & Dauter, 2011 ▸; Agrawal *et al.*, 2013 ▸).

Here, we look at fitting two tautomers of AMPPNP^4−^ into a 1.8 Å resolution crystal structure of the ATPase region of *Saccharomyces cerevisiae* topo­isomerase II (PDB entry 1pvg; Classen *et al.*, 2003 ▸; see also Agrawal *et al.*, 2013 ▸). A magnesium ion is observed next to the phosphates, so the AMPPNP is more likely to be in the 4− form than the 3− form. Two tautomers of AMPPNP^4−^ (Fig. 6 ▸) were drawn with *Marvin­Sketch*, and *MarvinSketch* was used to write out the corresponding SMILES strings (*Marvin* v.16.8.15, ChemAxon; https://www.chemaxon.com). The geometry of small-molecule crystal structures in the Cambridge Structural Database (CSD) containing PO_3_—NH—PO_3_, PO_3_—N=PO_3_ or PO_3_—N^−^—PO_3_ were examined manually (see Table 1 ▸ and Fig. 5 ▸) using *ConQuest* (Bruno *et al.*, 2002 ▸). It was observed that in small-molecule structures where two of the phosphate O atoms coordinate a divalent metal ion the bridging N atom was not protonated and the geometry was slightly different (data derived from such structures are indicated in Table 1 ▸).

The procedure we used was as follows.

(i) The AMPPNP was deleted from the coordinates of PDB entry 1pvg, and H atoms were added in *Coot* (*Coot*→Extension→Modelling→‘add H atoms using *REFMAC*’) and the structure was refined with *REFMAC* (Murshudov *et al.*, 2011 ▸). This should allow atoms in the protein to move to their ‘optimal’ positions without trying to ‘avoid’ clashes with the ligand.
(ii) Dictionaries and coordinates for the two tautomers of AMPPNP^4−^ (Fig. 6 ▸) were generated from the SMILES strings with *AceDRG* (Long *et al.*, 2017 ▸) and manually edited so that the geometry of the P—NH—P or P—N—P bonds was as in Table 1 ▸. [The problem of using Kekulé structures to describe delocalized bonds is well known (Katritzky *et al.*, 2010 ▸), and can cause problems for dictionary-making programs: in Supplementary Fig. S3 structures 8, 9 and 10 are different Kekulé representations of the same tautomer]. The analysis of the structures in the CSD suggested two types of dictionary for the unprotonated imino form (called P—N=P and P—N^−^—P in Table 1 ▸), with one dictionary for the imido (NH) form.
(iii) Coordinates for the ligand were real-space refined into the density (Fig. 6 ▸) in *Coot*, using all three dictionaries. Note that the real-space fit of the ligand was performed without coordinates for the protein, so that the ligand would try to optimally fit the density without ‘knowledge’ of the protein.
(iv) Ligand and protein files were combined in *Coot* and clashes checked for (*Coot* command Ligand→Isolated dots for Ligand), displaying only ‘bad overlaps’ in Fig. 6 ▸ (*Coot* command Draw→Generic display objects→Toggling off→Wide contacts, close contacts and H-bonds).
(v) Combined ligand and protein files were written out from *Coot* and refined with *REFMAC*.
(vi) Refined coordinates from the P—NH—P, P—N=P and P—N^−^—P ligands (Table 1 ▸) were read into *Coot* and the interactions were checked again (with Ligand→Isolated dots for this ligand *etc.*). The differences between the deposited structure in the PDB (1pvg), which does not have H atoms, and the three re-refined versions are small (Fig. 6 ▸ *e*).

With this AMPPNP example, the ‘correct’ answer appears to be the unprotonated N atom, because otherwise there is a clash between a backbone amide N atom and the N—H on the AMPPNP (Fig. 6 ▸). However, this ‘clash’ of H atoms can disappear in refinement, and is somewhat dependent on the lengths of the bonds to the H atoms (Deis *et al.*, 2013 ▸). In comparing a number of GHKL ATPase structures with AMPPNP, we noted (Agrawal *et al.*, 2013 ▸) that whereas some GHKL ATPase domain structures had the imido (P—N=P) form bound, others had the imido (P—NH—P) form bound. These observations prompted us to propose a mechanism for ATP hydrolysis in which the movement of a main-chain N—H past the bridging O atom in ATP causes it to protonate the bridging O atom, resulting in ATP hydrolysis (Supplementary Fig. S4; the ‘Wellington boot remover model’ of ATP hydrolysis).

### 5.2. Evaluating eight tautomers of QPT-1 (a spirocyclic barbituric acid) in a DNA–protein complex   

QPT-1 is a bacterial topoisomerase inhibitor that was discovered by Pharmacia in a whole-cell screen for compounds with antibacterial activity (Miller *et al.*, 2008 ▸). A compound derived from QPT-1, ETX0914 (formerly AZD0914), has completed a Phase 2 trial for the treatment of uncomplicated gonorrhoea and is due to go into a Phase 3 trial in 2017. The barbituric acid moiety of QPT-1 can adopt eight different tautomeric states (Supplementary Fig. S5).

**Figure 7:**
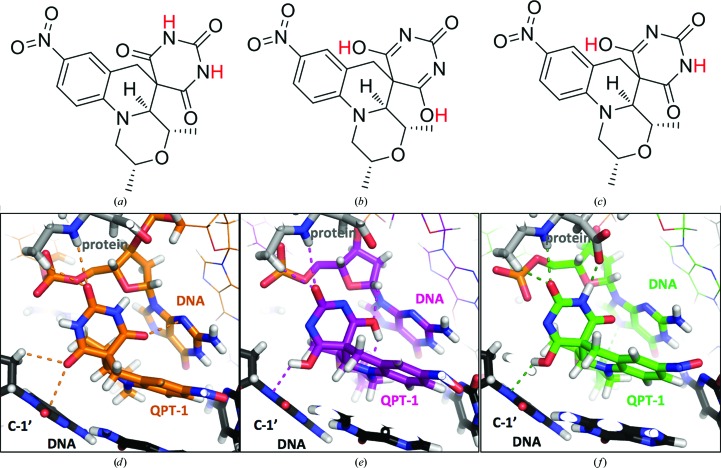
Three different tautomers of QPT-1 docked into three similar but slightly different binding sites in complexes of QPT-1 with DNA gyrase (protein) and DNA (for details, see Chan *et al.*, 2015 ▸).

To try to determine which tautomer was bound in each of six QPT-1 binding sites (from three DNA-cleavage complexes of *Staphylococcus aureus* DNA gyrase), we docked eight different tautomers into each of the six binding sites (Chan *et al.*, 2015 ▸). The program *AFITT* (Wlodek *et al.*, 2006 ▸), which can be run from the command line with a script, was used to dock and score the different possibilities. *AFITT* has three types of criteria for evaluating a docking pose: (i) a real-space correlation coefficient for the fit of the pose to the electron-density map, (ii) a ligand-strain score and (iii) two scores of the interaction between the ligand and the pocket, PLP and Chemscore. Because the eight different tautomers are quite similar, and because QPT-1 binds in the cleaved DNA making interactions with bases, we were not 100% certain which tautomer bound in which site. However, differences between the six QPT-1 binding sites suggested it was likely that different binding sites contained different QPT-1 tautomers (Fig. 7 ▸). Analysis of QPT-1 and other DNA complexes suggested that DNA gyrase ‘wriggles’ to effect the two DNA-cleavage and two DNA-religation steps in its catalytic cycle (Chan *et al.* 2015 ▸). It is not clear whether it is advantageous for compounds such as QPT-1 to be able to adopt different tautomers/shapes to maintain favourable interactions with their ligand-binding pocket as the pocket changes shape as the enzyme–DNA complex ‘wriggles’.

## 6. Experimental techniques to try to determine where your H atoms are   

X-rays are scattered by electrons and, as hydrogen has only one electron, hydrogen is seldom visible in a macromolecular X-ray crystal structure. Even in very high resolution X-ray crystal structures (1.2–0.65 Å) not all H atoms are visible in an electron-density map (Fisher *et al.*, 2012 ▸). A recent paper reviewing ‘Sub-atomic resolution X-ray crystallography and neutron crystallography’ (Blakeley *et al.*, 2015 ▸) stated that

While some details relating to H-atom positions are tractable with X-ray crystallography at sub-atomic resolution, the mobility of certain H atoms precludes them from being located. In addition, highly polarized H atoms and protons (H^+^) remain invisible with X-rays.

Electrons are charged particles and interact with both nuclei and electrons. A recent 1.4 Å resolution micro-electron diffraction study of crystals of the toxic core of α-synuclein (a short peptide) showed difference density for five out of a possible 73 protons at 2.8σ (Rodriguez *et al.*, 2015 ▸). However, high-resolution (better than 1.4 Å) electron diffraction is not yet easy to obtain and electrons, like X-rays, cause radiation damage.

Neutrons are scattered by nuclei, and the coherent scattering of neutrons by both hydrogen and its isotope deuterium is similar in size to the coherent scattering by other elements. Neutron crystallography is, in principle, the method of choice for experimentally determining the positions of H atoms (Blakeley *et al.*, 2015 ▸). In a 1.1 Å resolution neutron structure of crambin, 299 out of 315 (94.9%) of the H-atom positions were experimentally determined (Chen *et al.*, 2012 ▸). However, neutron crystallography remains technically challenging; in 2015 there were 83 macromolecular structures deposited in the PDB from neutron diffraction data, compared with more than 90 000 structures from X-ray data (Blakeley *et al.*, 2015 ▸). Neutron crystallography has been used to probe several reaction mechanisms in which protons or H atoms move, and has shown the presence of hydroxide (OH^−^) or hydronium (H_3_O^+^) ions (see, for example, Coates *et al.*, 2001 ▸; Kovalevsky *et al.*, 2010 ▸; Cuypers *et al.*, 2013 ▸; Casadei *et al.*, 2014 ▸).

NMR spectroscopy is probably the most popular technique for studying the tautomerism of small-molecule ligands in solution (Claramunt *et al.*, 2006 ▸). Its utility in determining the structural integrity of synthetic compounds that chemists rely so heavily on can be used to good effect to determine the experimentally found tautomeric states and their relative populations (see, for example, Zhu *et al.*, 2001 ▸). This allows the study of tautomeric equilibria and how factors such as pH, solvent and temperature can influence the most stable tautomers present. NMR studies are therefore as rich a source of information as computational studies of ligands alone. Unfortunately, when a ligand is bound to a protein the sheer number of NMR signals from the protein often swamp those from the ligand, making it much more difficult to extract the ligand information required to determine the bound tautomer. One way to get around this is by isotopically labelling the ligand with a low-abundance NMR-active isotope such as ^13^C or ^15^N or introducing an unusual NMR-active atom such as ^19^F into the ligand; this can help to filter out the protein signals and permit ligand-focused studies (Roberts, 1999 ▸).

## 7. Conclusions   

Since Watson & Crick (1953 ▸) ‘assumed that the bases only occur in the most plausible tautomeric forms’ structural and computational studies have shown that the four bases in DNA (G, C, A and T) do indeed each have only one stable tautomer (Saenger, 1983 ▸). Nevertheless, minor tautomeric forms of the DNA bases have been speculated to play a role in mutagenic mispairings during DNA replication (Topal & Fresco, 1976 ▸; Singh *et al.*, 2015 ▸), and in RNA biochemistry different tautomeric forms of bases can play important roles in the catalytic activity of ribozymes (Singh *et al.*, 2015 ▸). The transfer of protons (H^+^) or hydride ions (H^−^) clearly plays a key role in many reactions catalysed by enzymes (see, for example, the proposed mechanism for ATP hydrolysis in Supplementary Fig. S5), but definitively proving such mechanisms is challenging.

Although neutron crystallography (Blakeley, 2016 ▸) and other techniques can sometimes be used to determine the tautomeric state of a bound drug (Aggarwal *et al.*, 2016 ▸), for most routine X-ray crystal structures of protein–ligand complexes there will not be experimental evidence for the positions of the H atoms, and their positions must be inferred using chemical knowledge. The two examples presented in this paper show contrasting ease of determining the ‘correct’ tautomer. For some AMPPNP structures manual examination of crystal structures shows that hydrogen-bond donors point at the N atom between the β-phosphate and γ-phosphate, suggesting that it cannot be protonated. In contrast, in crystal structures of the antibacterial QPT-1 the docking and refinement of several different tautomers suggests that different QPT-1 molecules may adopt different tautomeric states as the compound-binding pocket changes shape, but exactly which tautomer is bound in each similar but slightly differently shaped pocket is not certain.

It has recently been reported that the refinement of high-resolution X-ray structures with a quantum-mechanical force field and the careful calculation of difference maps can help to determine which tautomer is bound (Borbulevych *et al.*, 2016 ▸). However, sometimes chemical common sense and careful evaluation of all possibilities may be all that is required.

## Supplementary Material

Supplementary Figures 1-5. DOI: 10.1107/S2059798316020283/ba5261sup1.pdf

## Figures and Tables

**Table 1:** Bond distances and angles for a P—NH—P or a P—N=P bond derived from the CSD The first numbers are from a manual analysis of the most closely related structures in the Cambridge Structural Database (*ConQuest* queried with PO_3_—N—PO_3_, where ‘any bond’ is allowed between P and O, and the P—N—P N atom is either NH or is not bonded to a third atom). The numbers in parentheses are the numbers of this type of bond (or angle) found in the CSD from which the information is derived. The numbers in bold are those given by *Mogul* (v.1.7.1), with the estimated standard deviation, when checking the structures. Note that the P1 and P2 atoms are not covalently bonded, but that the distance between these two P atoms depends on the P1—N—P2 bond angle, as well as the P—N bond lengths. Note that for the P—N=P angle *Mogul* only identified two examples, at 134 and 157°, giving a standard deviation of 16.3°. On a three standard deviation outlier score *Mogul* will allow P—N=P angles between 98 and 180°.

Atoms	P—NH—P (No.)	P—N= P (No.)	P—N^−^—P (No.)
P1—N (Å)	1.64 ± 0.01 (8)	1.59 ± 0.02 (3)	1.58 ± 0.01 (3)†
	**1.634 ± 0.028 (27)**	**1.578 ± 0.010 (32)**	**1.578 ± 0.010 (32)**
N=P2 (N—P2) (Å)	1.64 ± 0.01 (8)	1.53 ± 0.01 (3)	1.57 ± 0.02 (3)†
	**1.634 ± 0.028 (27)**	**1.528 ± 0.042 (16)**	**1.528 ± 0.042 (16)**
P1, P2 (Å)	2.97 ± 0.02 (4)	2.86 ± 0.04 (3)	2.78 ± 0.05 (3)†
P1—N—P2 (°)	130.0 ± 1.0 (4)	133.5 ± 3.0 (3)	124.0 ± 3.0 (3)†
	**129.859 ± 1.744 (9)**	**145.533 ± 16.323 (2)**	**145.533 ± 16.323 (2)**

† Two of the phosphate O atoms coordinate a divalent metal ion (see Fig. 5 ▸).
