# Supplementary material for: Getting the chemistry right: protonation, tautomers and the importance of H atoms in biological chemistry
Source: Acta Crystallogr D Struct Biol. 2017 Feb 1;73(Pt 2):131–40. doi: 10.1107/S2059798316020283 (PMC5297916; doi:10.1107/S2059798316020283)

STRUCTURAL
BIOLOGY

Volume 73 (2017)

Supporting information for article:

Getting the chemistry right: protonation, tautomers and the importance of hydrogens in biological chemistry

Supplementary Figure S1. (a1-a3) Three tautomeric forms of Viagra. (b1-b3) three tautomeric forms of glucose (Zhu et al., 2001). (c1-c5) five tautomeric forms of warfarin (Martin et al., 2009).

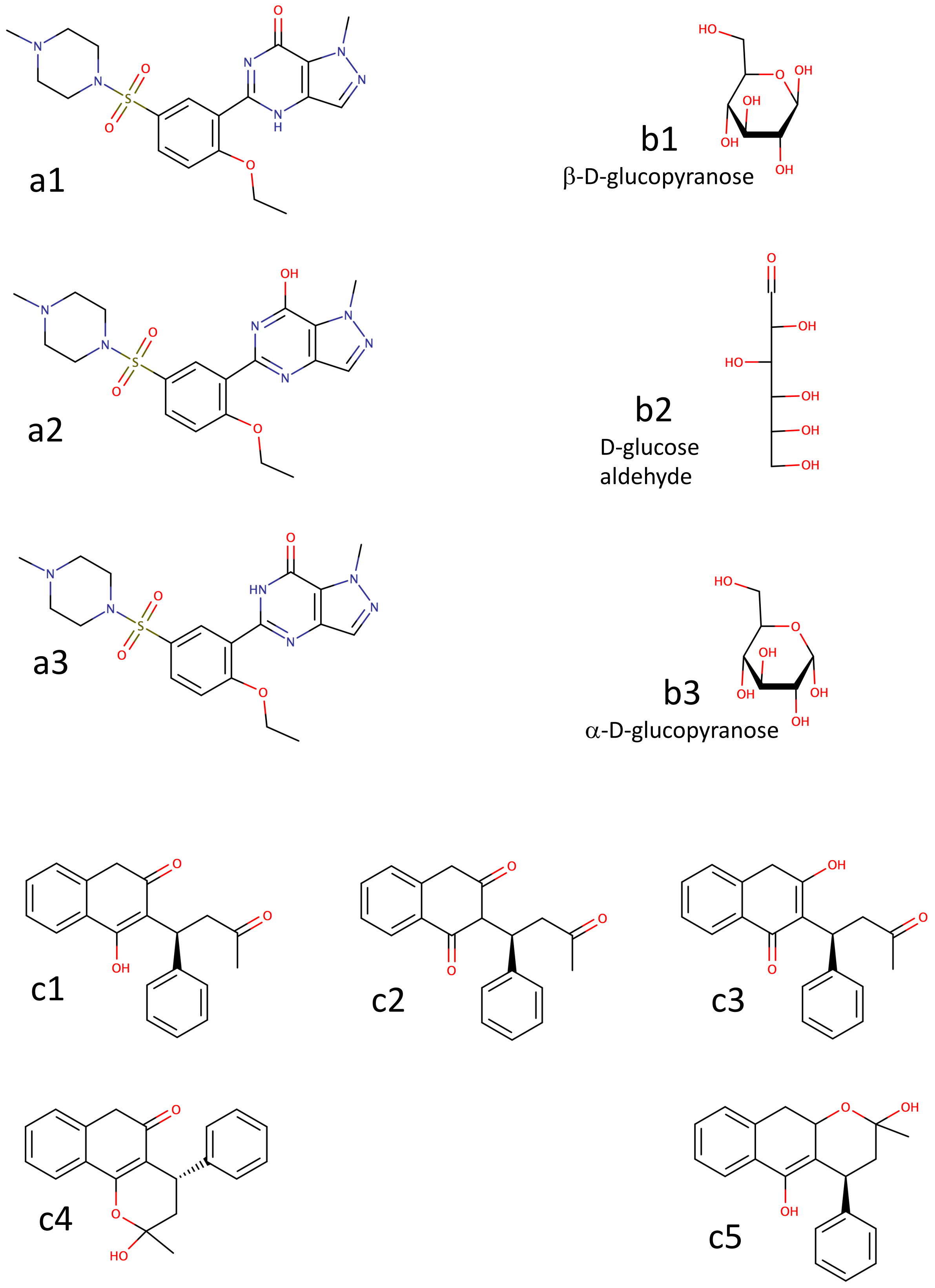

Supplementary Figure S2. Disputed structures from the CSD.
(**a**) The disputed tautomeric forms of 3-chloro-1,2,4-triazole.
(**b**) 2-amino-1,3-thiazolidin-4-one structures. Mogul highlights the GACXOZ exo-imino C=N bond length of 1.337 A as unusual, as the mean value in 50 equivalent structures is 1.289 A; also the C-N bond within the ring is rather short at 1.322 A, compared to a mean value of 1.364 A in 45 comparison structures. All the bond length, bond angle and dihedral angle values in the LOQBIE structure were considered to be typical. (Mogul February 2016 version used.) **(c)** The disputed forms of a 2-amino-1,3,4-thiadiazole. Bond lengths highlighted by Mogul as unusual are shown in red. UKIRAI also has 4 unusual bond angles (thiadiazole S-C-N, sulfonamide O-S-N, C-S-N, and O-S-C) and 2 torsions (sulfonamide O-S-C-N and C-S-N-C), whereas UKIRAI02 has 3 bond angles (thiadiazole C-S-C, C-N-N, and S-C-N) and 2 torsions (sulfonamide O-S-C-N and C-S-N-C).

a

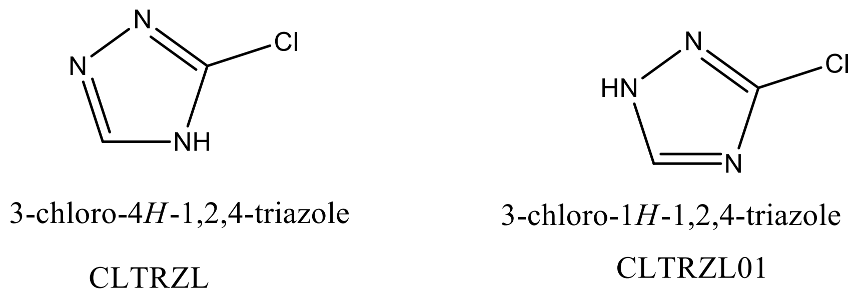

b

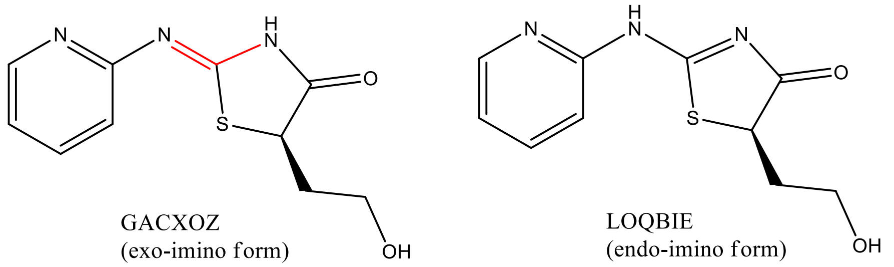

c

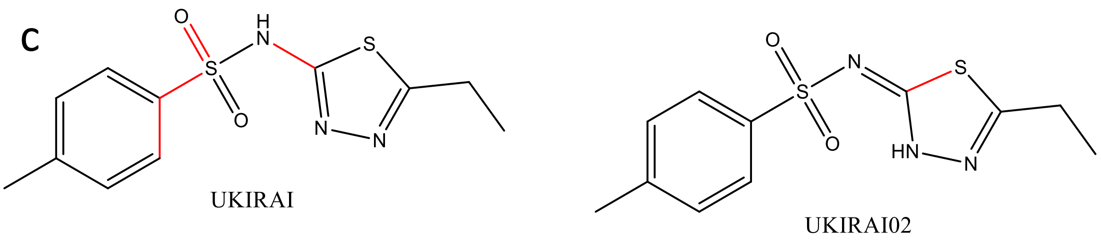

Supplementary Figure S3. Kekule representations of the phosphates in ATP^{3-}(1), ATP^{4-}(2), AMPPNP^{3-}(3-5), and AMPPNP^{4-} (6-10). Note that structures 8, 9 and 10 are different resonance structure of the same tautomer. *Marvin* was used for drawing chemical structures (Marvin, 2016).

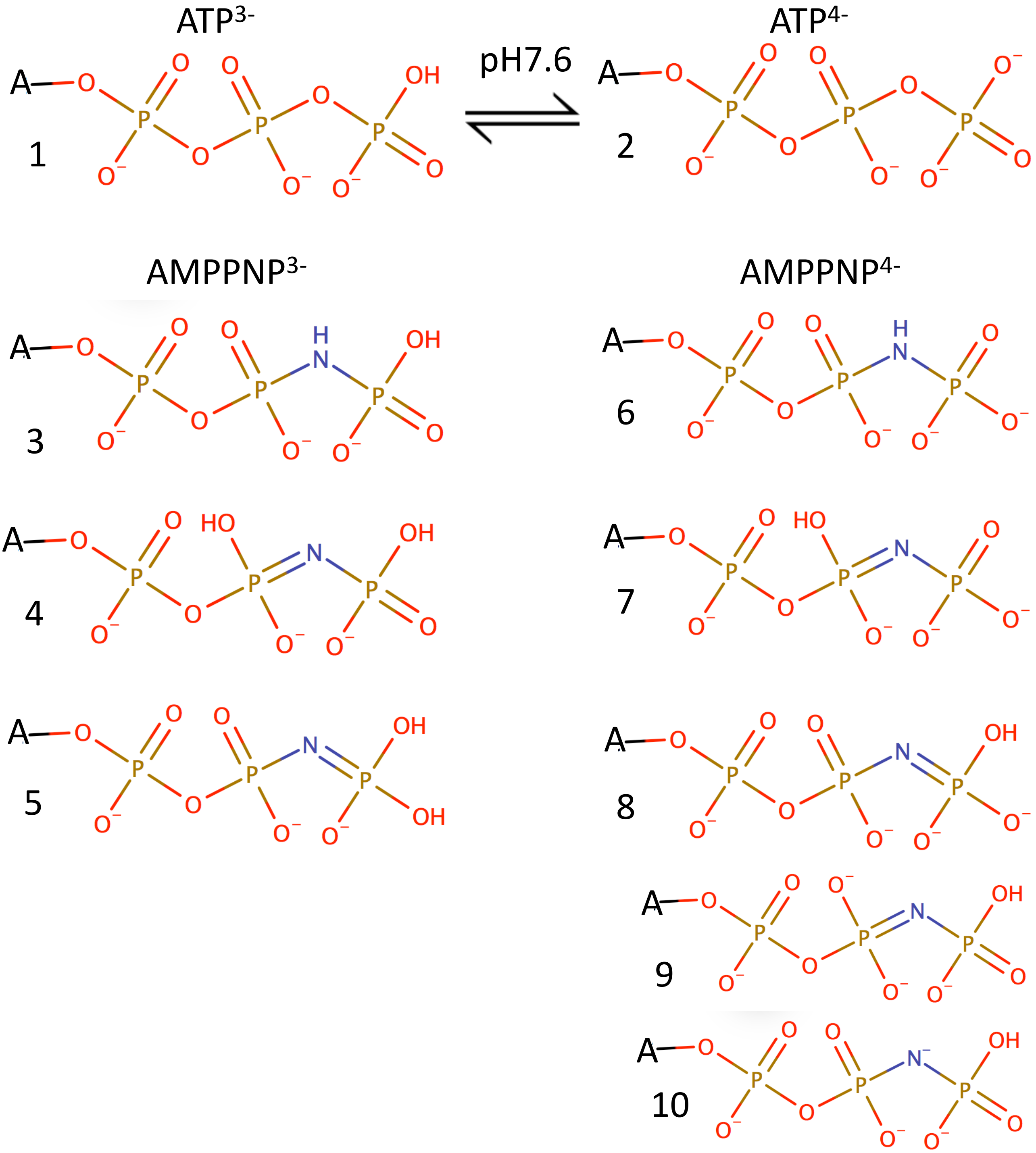

Supplementary Figure S4. The 'Wellington boot remover' model for ATP hydrolysis in the GHKL ATPase domains of type IIA topoisomerases. The movement of the main-chain N-H past the bridging oxygen causes it to leave its proton behind.

a

A backbone amide NH is moved past the oxygen between the beta and gamma phosphates.

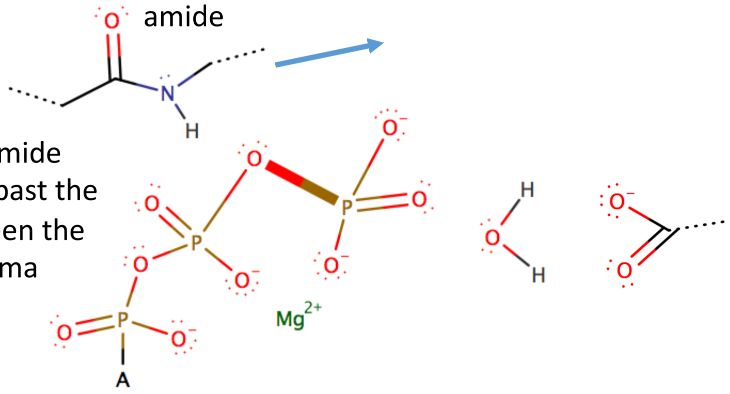

b

The amide has protonated the bridging oxygen, producing a highy reactive metaphosphate ion [PO_3^-].

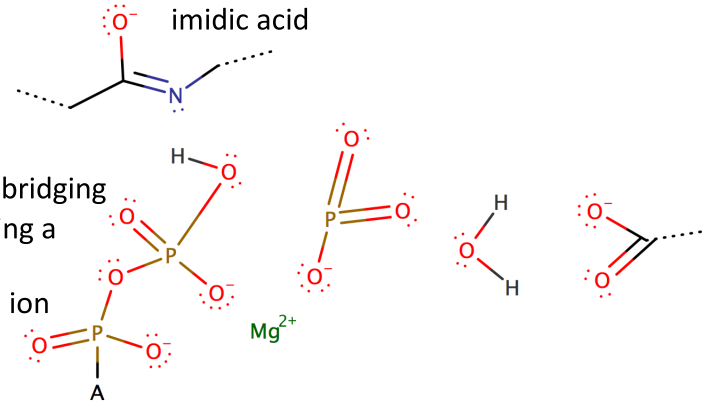

c

The metaphosphate ion [PO_3^-] has reacted with a correctly position 'attacking water'

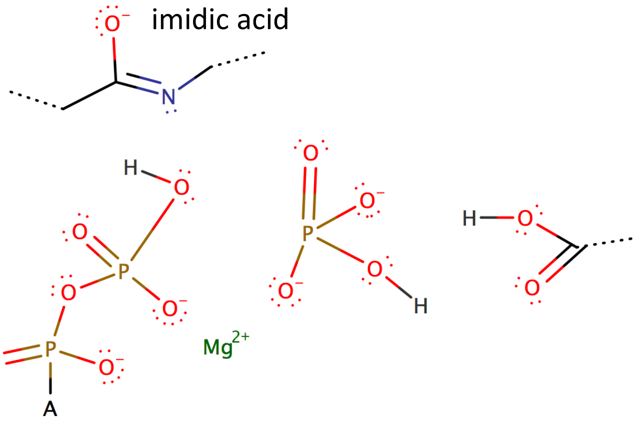

c

The ADP^{2-} ion has given a hydrogen back to the amide, and the catalytic Glu has give a hydrogen to the phosphate ion.

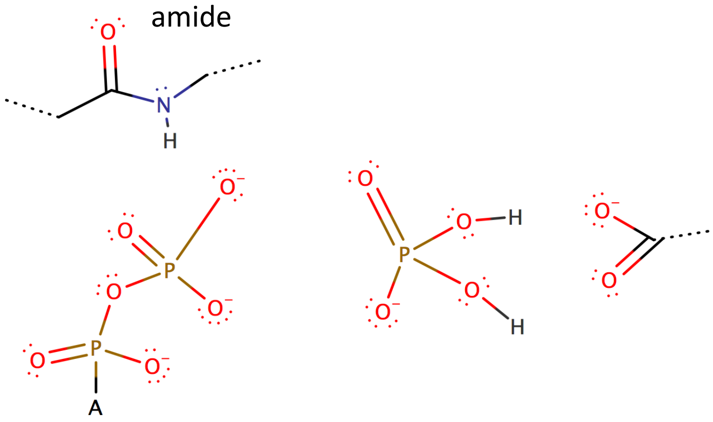

Supplementary Figure S5. Eight tautomeric forms of QPT-1.

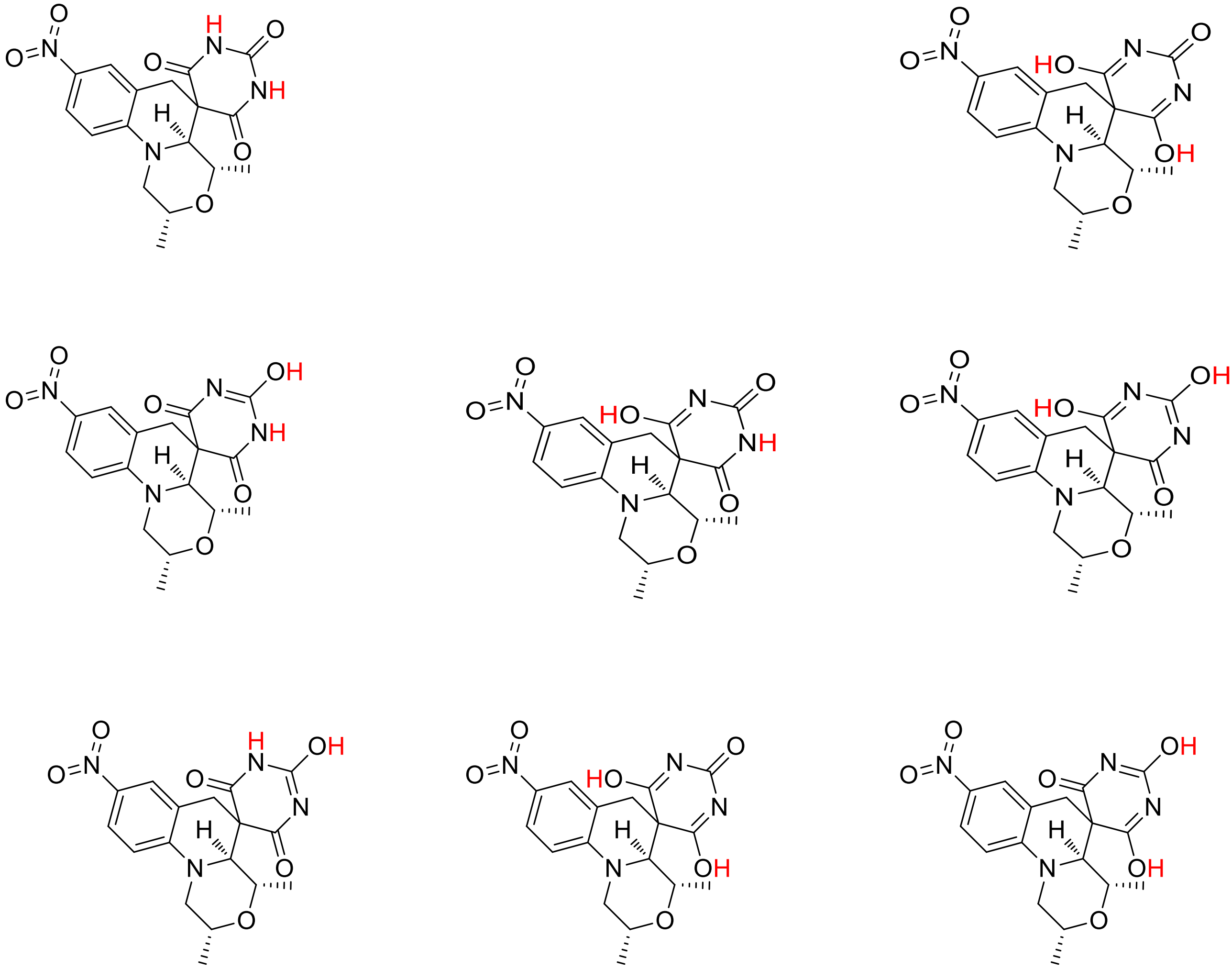

Supplement: Supplementary file 1 [file d-73-00131-sup1.pdf]
